# Glucocorticoids increase skeletal muscle NF‐*κ*B inducing kinase (NIK): links to muscle atrophy

**DOI:** 10.14814/phy2.13014

**Published:** 2016-11-14

**Authors:** Christopher S. Fry, Syed Z. Nayeem, Edgar L. Dillon, Partha S. Sarkar, Batbayar Tumurbaatar, Randall J. Urban, Traver J. Wright, Melinda Sheffield‐Moore, Ronald G. Tilton, Sanjeev Choudhary

**Affiliations:** ^1^ Department of Nutrition and Metabolism The University of Texas Medical Branch (UTMB) Galveston Texas; ^2^ Department of Internal Medicine Division of Endocrinology The University of Texas Medical Branch (UTMB) Galveston Texas; ^3^ Department of Neurology The University of Texas Medical Branch (UTMB) Galveston Texas

**Keywords:** Atrogin‐1, catabolism, methylprednisolone, MuRF1, NF‐*κ*B inducing kinase

## Abstract

Glucocorticoids (GC) are a frontline therapy for numerous acute and chronic diseases because of their demonstrated efficacy at reducing systemic inflammation. An unintended side effect of GC therapy is the stimulation of skeletal muscle atrophy. Pathophysiological mechanisms responsible for GC‐induced skeletal muscle atrophy have been extensively investigated, and the ability to treat patients with GC without unintended muscle atrophy has yet to be realized. We have reported that a single, standard‐of‐care dose of Methylprednisolone increases in vivo expression of NF‐*κ*B‐inducing kinase (NIK), an important upstream regulatory kinase controlling NF‐*κ*B activation, along with other key muscle catabolic regulators such as Atrogin‐1 and MuRF1 that induce skeletal muscle proteolysis. Here, we provide experimental evidence that overexpressing NIK by intramuscular injection of recombinant human NIK via adenoviral vector in mouse tibialis anterior muscle induces a 30% decrease in the average fiber cross‐sectional area that is associated with increases in mRNA expression of skeletal muscle atrophy biomarkers MuRF1, Atrogin‐1, myostatin and Gadd45. A single injection of GC induced NIK mRNA and protein within 2 h, with the increased NIK localized to nuclear and sarcolemmal locations within muscle fibers. Daily GC injections induced skeletal muscle fore limb weakness as early as 3 days with similar atrophy of muscle fibers as observed with NIK overexpression. NIK overexpression in primary human skeletal muscle myotubes increased skeletal muscle atrophy biomarkers, while NIK knockdown significantly attenuated GC‐induced increases in NIK and Atrogin‐1. These results suggest that NIK may be a novel, previously unrecognized mediator of GC‐induced skeletal muscle atrophy.

## Introduction

Since the advent of glucocorticoid (GC) therapy for the treatment of autoimmune diseases, the use of steroids to treat disease has grown steadily so that today glucocorticoids are used in virtually every subspecialty of medicine. Approximately, 2.5 million adults in the United States require oral glucocorticoid therapy for a variety of inflammatory conditions, and nearly one‐third of these individuals are treated for periods exceeding 5 years (Overman et al. 2013). This therapy is the front‐line treatment for a large number of acute and chronic inflammatory and neurological pathologies, including myositis (Dalakas 2015; Moghadam‐Kia et al. 2015), asthma (Kelly et al. 2012), chronic obstructive pulmonary disease (Walters et al. 2014), rheumatoid arthritis (Pincus 2011), psoriasis (Ash et al. 2012; Gustafson et al. 2013), inflammatory bowel disease (De Iudicibus et al. 2011), multiple sclerosis (Burton et al. 2012), and spinal cord injury (Qian et al. 2005; Bracken 2012), and is widely used following organ transplant surgery to prevent rejection and in cancer treatment to reduce chemotherapy side effects. The well‐recognized clinical benefits of glucocorticoids are based on their excellent anti‐inflammatory and immunosuppressive properties, but the risk for significant side effects is equally appreciated. These include hypertension, osteoporosis, central obesity, dyslipidemia, steroid‐induced insulin resistance leading to diabetes, skeletal muscle atrophy, and ultimately, decreased survival (Schacke et al. 2002; Huscher et al. 2009; Geer et al. 2014; van Raalte and Diamant 2014).

While the risk of significant muscle atrophy with GC therapy is widely appreciated (Khaleeli et al. 1983; Mills et al. 1999; Carroll and Findling 2010; Hanaoka et al. 2012), the primary cause of this glucocorticoid effect in skeletal muscle remains the focus of intensive investigation. This muscle atrophy is best appreciated in humans with Cushing syndrome where endogenous glucocorticoids are overproduced and skeletal muscle mass is greatly reduced compared with weight‐matched controls (Geer et al. 2010). Other highly relevant examples occur when treating cancer or in other acute/chronic inflammatory conditions in middle‐aged to older adults where the physiologic tolerance for any degree of muscle loss is low, and even modest losses can quickly impact morbidity and mortality.

Based on clinical and basic science observations from our laboratories, we have demonstrated that a key, upstream kinase involved in NF‐*κ*B (nuclear factor‐kappa B) pathway activation NIK (NF‐*κ*B Inducing Kinase) ‒ is highly expressed in skeletal muscle in response to GC therapy in humans (Urban et al. 2014). We have shown that a single, standard‐of‐care dose of GC increases in vivo and in vitro expression of NIK along with skeletal muscle atrogenes, Atrogin‐1 and MuRF1 (muscle RING‐finger protein‐1), that induce skeletal muscle proteolysis (Urban et al. 2014). NIK acts as a proximal inducer of the I*κ*B kinase complex, an upstream convergent point for numerous signals leading to NF‐*κ*B activation (Lin et al. 1998; Xiao et al. 2001; Dejardin et al. 2002; Bonizzi et al. 2004). Although all cells continually express this kinase, its expression is regulated at a posttranslational level to maintain very low basal steady‐state levels. Potential molecular mechanisms responsible for GC‐induced increases in NIK expression and the impact of elevated NIK levels on the progression of muscle atrophy are not known. Here, we have utilized animal models to demonstrate that GC induces NIK mRNA and protein expression, and that both systemic GC administration and NIK overexpression in tibialis anterior (TA) muscle induce a significant shift to smaller muscle fiber size and decrease overall myofiber cross‐sectional area (CSA). Additionally, increased NIK expression is localized to the smaller muscle fibers. We also utilize human skeletal muscle myotubes in tissue culture to demonstrate that overexpressing NIK increased atrogene expression, and silencing NIK prior to GC exposure significantly decreases the extent of muscle atrogene expression induced by GC. These results provide support for the hypothesis that increased skeletal muscle NIK levels are associated with, and may modulate GC‐induced muscle atrophy.

## Materials and Methods

### Human studies

#### Human skeletal muscle biopsy

The study utilized healthy male human skeletal muscle obtained from a clinical study approved by The University of Texas Medical Branch Institutional Review Board and complied with the Declaration of Helsinki. Written informed consent was obtained from all subjects. Skeletal muscle tissue was collected from the lateral portion of the vastus lateralis, using a 5 mm Bergström biopsy needle as previously described (Urban et al. 2014; Hernandez‐Jimenez et al. 2012). These muscle biopsies were used to generate the differentiated myotubes used in the tissue culture studies detailed below.

### Animal studies

#### Animals

All animal work was conducted in accordance with an Institutional Animal Care and Use Committee (IACUC)‐approved protocol, the Public Health Service *Guide for the Care and Use of Laboratory Animals*, and the UTMB Institutional Biosafety Committee's Notice of Use approval for the adenoviral recombinant DNA work. Male C57BL/6 mice were purchased at 6–8 weeks of age, group housed (5/cage) in the UTMB Animal Resource Center, and fed standard food and water ad libitum with a 12/12 h light/dark cycle.

#### Glucocorticoid experiments

Wild‐type C57BL/6 mice were randomized to methylprednisolone (Medrol) (100 mg/kg injected s.c.; *n* = 4–6) or sham (phosphate‐buffered saline [PBS]; *n* = 4), then euthanized 2 and 4 h later with an anesthetic overdose, and both tibialis anterior muscles were isolated and separately processed for qRT‐PCR, Western blotting, and immunohistochemistry. USP grade methylprednisolone (Sigma, St. Louis, MO, cat# M0639) was first dissolved in DMSO, then diluted to final concentration, using polyethylene glycol. In additional experiments, wild‐type C57BL/6 mice were injected s.c. once daily for 10 days with 10 mg/kg methylprednisolone (*n* = 5), while sham controls (*n* = 5) were injected with sterile PBS. Changes in muscle function were tracked longitudinally at baseline then every 3rd day after initiating methylprednisolone, using a grip strength test (Harvard Apparatus, Holliston, MA) of both paws of fore limbs (Fry et al. 2015). Mice were euthanized with an anesthetic overdose, and tibialis anterior muscles were dissected, freed from connective tissue, weighed, and mounted in optimal cutting temperature (OCT) compound at resting length for determination of fiber CSA.

#### NIK overexpression experiments

Following transfer of animals into the UTMB ARC animal biosafety level 2 space, mice (*n* = 8) were anesthetized with ketamine/diazepam (70–90/10 mg/kg intraperitoneal [i.p.]), and 1 × 10^11^ pfu (plaque‐forming units) AAV‐NIK was injected intramuscularly into the tibialis anterior (TA) in a total volume of 50 μL of sterile phosphate‐buffered saline. As a control, an AAV vector containing green fluorescence protein (GFP) was injected (1 × 10^11^ pfu in 50 μL) into the tibialis anterior on the contralateral leg. Initial experiments assessed the optimal duration for increased NIK expression; our data indicated that at least 3 weeks was necessary for significant increases in human NIK expression in the mouse skeletal muscle, and this time interval was used in our subsequent experiments. Following euthanasia, both tibialis anterior muscles were removed and either frozen in liquid nitrogen and stored at −80°C for subsequent protein and RNA extraction, or were embedded in OCT for histology and immunofluorescence studies as detailed below.

#### Preparation of an adeno‐associated virus‐expressing NIK cDNA

An updated triple plasmid transfection protocol was used to generate recombinant AAV vectors. Full length NIK cDNA was cloned into the large capacity *cis‐*plasmid pAAV‐MCS (Agilent Technologies, Santa Clara, CA). After confirming the sequence integrity of the clones and the inverted terminal repeats in the plasmid, the plasmids were amplified for viral preparation, using Qiagen endotoxin‐free plasmid DNA purification kit. All other plasmids used for adeno‐associated virus (AAV) production in this study were obtained from Vigene Biosciences (Rockville, MD). The AAV vector production was also performed by Vigene Biosciences. Briefly, the transfection mixture contained (1) the pHelper plasmid; (2) pRC plasmid containing *cap and rep* genes; and (3) pAV‐NIK, flanked by inverted terminal repeats derived from the AAV2 genome. Vector purification was carried out using iodaxinol gradient ultracentrifugation protocol, buffer exchange, and concentration using Amicon Ultracel‐15 with 100 kDa molecular weight cut‐off (MWCO) centrifugation columns (EMD Millipore, Billerica, MA). AAV physical titers were obtained by quantitative PCR (iCycler, BioRad, Hercules, CA), using primers designed to selectively bind AAV2 inverted terminal repeats (forward, 5′‐ GACCTTTGGTCGCCCGGCCT ‐3′; reverse, 5′‐ GAGTTGGCCACTCCCTCTCTGC ‐3′).

### Tissue culture

#### Primary human skeletal muscle myoblast isolation and differentiation

Fresh tissue for primary cell culture was collected in ice‐cold transport media (Ca^2+^ and Mg^2+^‐free Hanks Balanced Salt Solution; Invitrogen, Carlsbad, CA) and immediately processed (Urban et al. 2014; Hernandez‐Jimenez et al. 2012). Briefly, fresh skeletal muscle biopsy samples (0.05–0.2 g) were carefully teased apart separating skeletal muscle from fat and blood clots, minced finely, and then digested by dispase II and collagenase for 40 min at 37°C. The dispersed cells were filtered through 40 μm cell strainer, centrifuged, and suspended in growth medium (Hams F10 media + 20% fetal bovine serum + bFGF (fibroblast growth factor; 5 ng/mL final) + 1% penicillin/streptomycin + 0.1% gentamicin). The cell suspension was transferred to a 60 mm culture dish and left undisturbed for 48–72 h in a tissue culture incubator, with fresh media added every 3 days thereafter. The cells were differentiated for 3–5 days by culturing them in differentiation medium (minimum essential medium eagle alpha Modification‐containing 1% penicillin/streptomycin, 0.1% gentamicin, and 2% horse serum).

#### NIK overexpression

The eukaryotic expression vector pRK‐MycNIK was described previously by Woronicz et al. (1997). Plasmids were purified by ion exchange (QIAGEN, Chatsworth, CA) and sequenced to verify authenticity. After 4 days of differentiation, primary human skeletal muscle myotubes were transfected with increasing concentrations of NIK wild‐type (NIKwt) expression vector (0, 0.25, 0.5 and 1 μg/mL) by FuGENE‐6 transfection reagent (Promega; Madison, WI), using the manufacturer's protocol. After 48 h, RNA was extracted by Trizol reagent (Sigma) and mRNA (messenger ribonucleic acid) expression of muscle catabolic genes including FOXO1 (forkhead box O1), REDD1 (regulated in development and DNA damage responses 1), Atrogin‐1, MuRF‐1, and elF4EBP1 (eukaryotic translation initiation factor 4E‐binding protein 1) was measured by quantitative real‐time polymerase chain reaction (qRT‐PCR) as detailed below.

#### NIK knockdown

Four days after initiating the differentiation protocol, primary cultures of human skeletal myotubes were transfected with either NIK‐specific ON‐TARGETplus l siRNA or validated nontargeting negative control siRNA (GE Dharmacon, Lafayette, CO) at a concentration of 50 nmol/L for 72 h followed by exposure to methylprednisolone (0.5 μg/mL) for an additional 24 h. RNA was extracted with Trizol reagent, and the extent of NIK mRNA knockdown determined by qRT‐PCR, using glyceraldehyde‐3‐phosphate dehydrogenase (GAPDH) as the housekeeping gene for data normalization as detailed below.

### Quantitative real‐time PCR (qRT‐PCR)

Total cellular RNA was extracted from mouse skeletal muscle and human myotubes by Tri Reagent (Sigma). RNA was quantified, using Nanodrop 2000 spectrophotometer (Thermo Scientific, Waltham, MA). A 260/280 ratio was used to verify the quality of RNA, and RNA preparations with a ratio between 1.8 and 2.0 were used for cDNA synthesis. 2 μg of RNA was used for reverse transcription using the SuperScript III First‐Strand Synthesis System from Invitrogen (Carlsbad, CA). 2 μL of cDNA products were amplified in a 20‐μL reaction system containing 10 μL of iQ SYBR Green Supermix (Bio‐Rad) and 400 nmol/L primer mixture. Relevant primers were purchased from SA Bioscience, (Frederick, MD); exact sequences are proprietary to the company and not available for publication. All reactions were processed in a MyiQ Single Color Real‐Time PCR thermocycler, using a two‐step plus melting curve program, and the results were analyzed by the iQ5 program (Bio‐Rad). For each gene, mRNA expression was calculated with the delta delta Ct/quantitative method, with results normalized to GAPDH as a housekeeping gene and expressed as fold change relative to its expression in the absence of NIK transfection. Human NIK expression in mouse skeletal muscle is reported as a decrease in cycle times (fewer cycle times implies more starting RNA in the sample) since it is absent in the control mouse skeletal muscle overexpressing only GFP.

To assess the involvement of protein catabolism in skeletal muscle resulting from overexpression of NIK, Atrogin‐1, MuRF‐1, myostatin, Gadd45 (growth arrest and DNA damage‐inducible 45), and MyoD (myogenic differentiation 1) gene expression was measured in mouse skeletal muscle tissue extracts by qRT‐PCR. To assess the effect of NIK overexpression on expression of catabolism markers, Atrogin‐1, MuRF‐1, elF4EBP1, FOXO1, and REDD1 gene expression was measured in primary myotube extracts by qRT‐PCR.

### Immunohistochemistry

Immunohistochemistry was performed 2 and 4 h after exposure to a single dose of methylprednisolone and 3 week following injection of AAV‐GFP or AAV‐NIK. Tibialis anterior (TA) muscles were removed from anesthetized mice, pinned to a cork block at resting length, covered with a thin layer of Tissue Tek optimal cutting temperature (OCT) compound (Sakura Finetek, Torrance, CA), quickly frozen in liquid nitrogen‐cooled isopentane, and stored at −80°C until sectioning. Frozen muscles were sectioned (7 μm), air‐dried for at least an hour and stored at −20°C. Sections were stained with hematoxylin and eosin following standard protocols. Slides were mounted with cytoseal XYL (Thermo Fisher Scientific, #8312‐4).

For NIK detection, slides were fixed in 4% paraformaldehyde, followed by epitope retrieval using sodium citrate (10 mmol/L, pH 6.5) at 92°C for 20 min. Sections were blocked for 1 h with 2.5% normal horse serum (Vector Laboratories, Burlingame, CA, #S‐2012) and Mouse‐on‐Mouse Blocking Reagent (Vector Laboratories, #MKB‐2213). Sections were then incubated with anti‐dystrophin (Vector Laboratories, #VP D505) and anti‐NIK (Cell Signaling, Danvers, MA, #4994) overnight at 4°C. Sections were washed with PBS and then incubated with species‐specific secondary antibodies (Life Technologies, Carlsbad, CA, #A‐11001 and A‐21429). Sections were subsequently washed with PBS and co‐stained with DAPI (10 nmol/L, Life Technologies, #D35471) prior to being mounted with anti‐fade media (Vector Laboratories, #H‐1000).”

For GFP detection, slides were fixed in 4% paraformaldehyde, and then sections were blocked for 1 h with 2.5% normal horse serum (Vector Laboratories, #S‐2012) and Mouse‐on‐Mouse Blocking Reagent (Vector Laboratories, #MKB‐2213). Sections were then incubated with anti‐dystrophin and anti‐GFP (Abcam, Cambridge, MA, #ab13970) overnight at 4°C. Sections were washed with PBS and then incubated with species‐specific secondary antibodies (Life Technologies, #A‐11001 and A‐21467). Sections were subsequently washed with PBS and co‐stained with DAPI prior to being mounted with anti‐fade media.

Slides were imaged at 20× magnification at room temperature with a Zeiss upright microscope (AxioImager M1; Carl Zeiss, Oberkochen, Germany), and analysis was carried out using the AxioVision Rel 4.9 software (Zeiss, Oberkochen, Germany). Individual fibers were traced to determine cross‐sectional area. To capture the entire tibialis anterior muscle cross section required analysis of 8–12 images (average number of fibers analyzed: 568 [AAV‐GFP]; 553 [AAV‐NIK]; 462 (PBS); 563 (methylprednisolone)). To determine cross‐sectional area of NIK+ fibers, fibers‐expressing perinuclear NIK staining in a nucleus residing completely within the dystrophin border were scored as NIK+. Fibers with no visible myonuclear NIK staining were scored as NIK−. GFP+ fibers were scored as GFP+ with ubiquitous expression of GFP within the muscle fiber.

### Statistical methods

All results are expressed as mean ± SD. Where indicated, two‐way independent and repeated measures ANOVA tests were performed to evaluate overall group differences followed by the Holm‐Sidak post hoc test to determine pair‐wise significance. In the case of only two group comparisons, a two‐sample Student's *t*‐test was performed with either equal or unequal variance after checking for variance distribution via Levene's test. Differences in fiber cross‐sectional area were determined via a paired student's *t*‐test, as AAV‐GFP‐ and AAV‐NIK‐treated muscle was collected from contralateral limbs in the same mouse. In all cases, *P < *0.05 was considered significant.

## Results

### Methylprednisolone increases mouse skeletal muscle NIK

**Figure 1:**
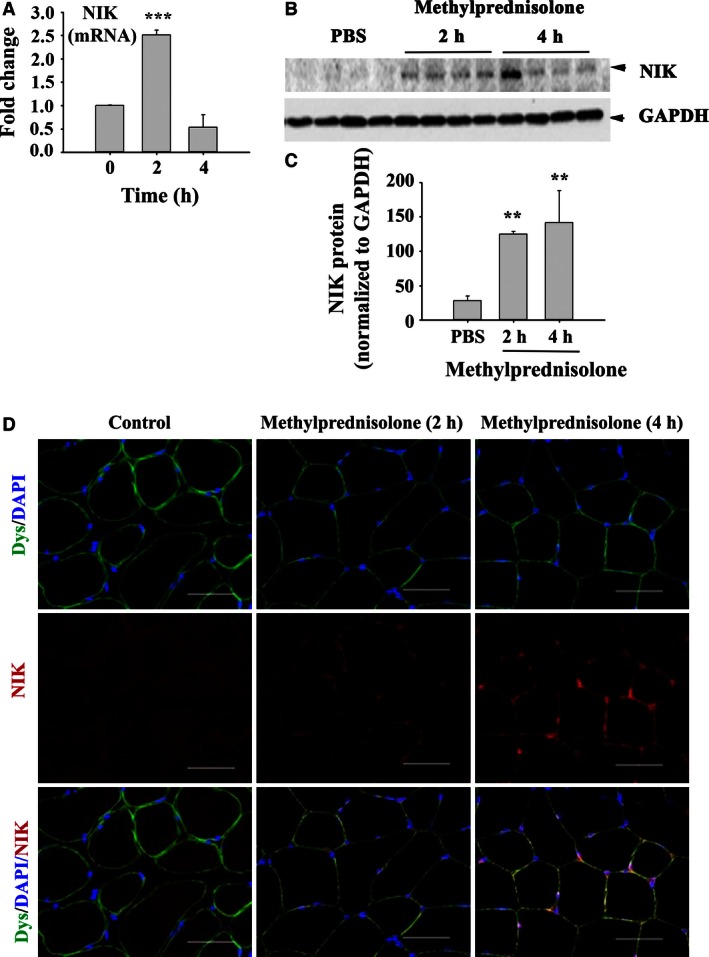
Methylprednisolone administration induces acute elevations in NIK mRNA and protein expression. (A) *NIK* mRNA levels presented as a fold change from control following a single, subcutaneous injection of 100 m g/kg body weight methylprednisolone. (B) Quantification of NIK protein levels following methylprednisolone administration using western blot. (C) Representative immunohistochemical images depicting dystrophin (green), NIK (red) and DAPI (blue) in cross‐sections of the tibialis anterior following methylprednisolone administration. Scale bar = 50 μm. Data are presented as mean ± SD. Significant effect of methylprednisolone administration, **P < 0.01 and ***P < 0.001. *N* = 4–6 mice/group.

**Figure 2:**
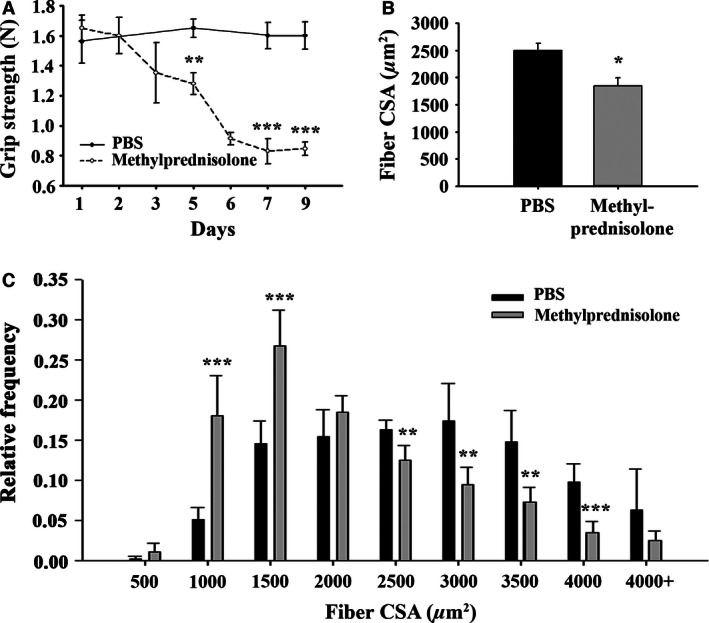
Chronic methylprednisolone administration induces muscle weakness and atrophy. (A) Average forelimb grip strength in *N*. (B) Average myofiber cross‐sectional area (CSA) in μm^2^. (C) Histogram distribution of myofiber CSA. Data are presented as mean ± SD. ** Significantly effect of methylprednisolone administration: *P < 0.05, **P < 0.01 and ***P < 0.001. *N* = 5 mice/group.

We have reported previously that NIK mRNA levels quantified by qRT‐PCR were significantly elevated in human skeletal muscle biopsy specimens after a 6‐day, graded dosing regimen of methylprednisolone (24, 20, 16, 12, 8, and 4 mg on days 1 through 6, respectively), and that methylprednisolone (0.5 μg/mL)‐induced NIK protein expression in differentiated C2C12 cells (Urban et al. 2014). Here, we report that methylprednisolone induces NIK message and protein in skeletal muscle of wild‐type C57BL/6 mice. NIK mRNA and protein levels were increased within 2 h in tibialis anterior muscle following a single subcutaneous injection of 100 mg/kg body weight methylprednisolone (Fig. 1A and B). While NIK mRNA decayed to below baseline levels by 4 h, protein levels assessed with Western blotting (Fig. 1B) and immunohistochemistry (Fig. 1C) remained elevated through 4 h postinjection. The pattern of NIK staining shown in Figure 1C is predominantly peri‐nuclear and along the internal aspect of the sarcolemmal membrane in a punctate pattern. Daily subcutaneous injection of 10 mg/kg body weight methylprednisolone significantly impaired skeletal muscle function assessed using grip strength testing within 3 days of initiating treatment (Fig. 2A) and produced a significant decrease in average muscle fiber CSA (Fig. 2B) and a shift to smaller fiber size (Fig. 2C) in tibialis anterior muscle after 10 days of treatment.

### NIK overexpression in skeletal muscle is associated with muscle atrophy

**Figure 3:**
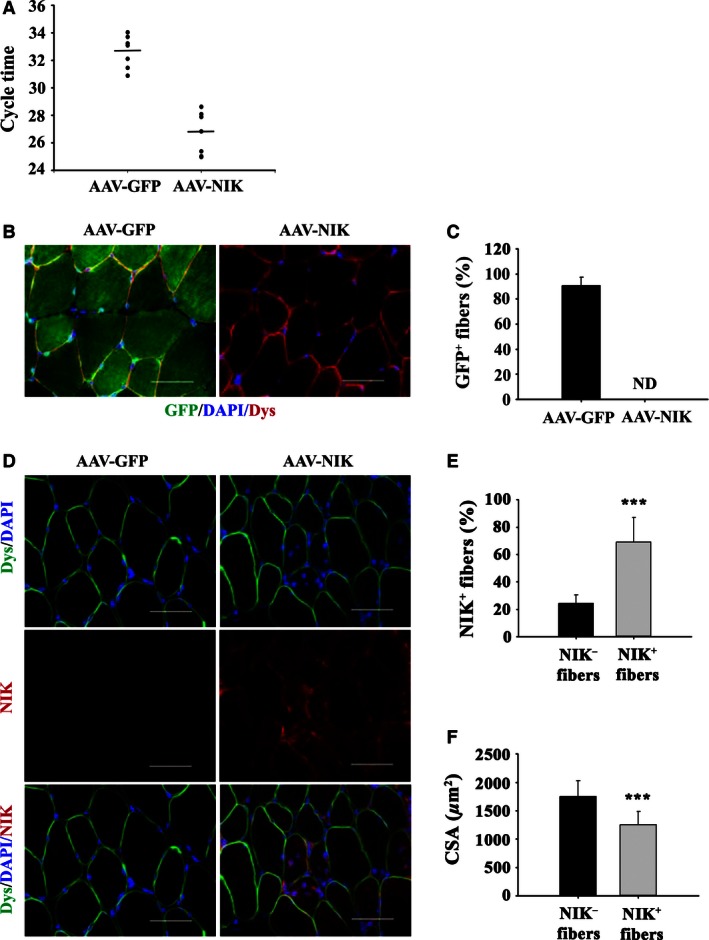
Overexpressing human NIK in mouse tibialis anterior induces muscle atrophy. (A) NIK mRNA levels presented as cycle times three weeks after injection of AAV‐NIK or AAV‐GFP. (B) Representative immunohistochemical images denoting dystrophin (red), GFP (green) and DAPI (blue) in cross‐sections of the tibialis anterior following administration of AAV‐NIK or AAV‐GFP. Scale bar = 50 μm. (C) Percentage of fibers‐expressing GFP presented as mean ± SD. ND, none detected. (D) Representative immunohistochemical images denoting dystrophin (green), NIK (red) and DAPI (blue) in cross‐sections of the tibialis anterior following administration of AAV‐NIK or AAV‐GFP. Scale bar = 50 μm. (E) Percentage of fibers‐expressing NIK perinuclear staining presented as mean ± SD. (F) Average myofiber cross‐sectional area (CSA) in μm^2^ of NIK− and NIK+ fibers following administration of AAV‐NIK. Data are presented as mean ± SD. ***Significant effect of AAV‐NIK administration, *P* < 0.001. *N* = 8 mice/group.

To explore further a direct effect of NIK on skeletal muscle atrophy in vivo, we performed initial experiments that determined a time‐course of human NIK expression in the mouse tibialis anterior muscle following injection of AAV‐NIK. Significant increases in NIK mRNA shown as lower PCR cycle times versus background (the AAV‐GFP muscle contains no human NIK) were observed after 3 weeks (Fig. 3A). The GFP protein product of the control AAV‐GFP was present throughout the cytoplasm (Fig. 3B) in approximately 90% of all muscle fibers, indicating robust infection (Fig. 3C). Similar to the methylprednisolone‐induced NIK protein localization shown in Figure 1C, AAV‐NIK‐induced NIK expression localized to the peri‐nuclear and peri‐sarcolemmal regions of the muscle fibers (Fig. 3D). Approximately 70% of the muscle fibers stained positive for NIK (Fig. 3E), which likely represents an underestimation as not all fibers contained a visible myonucleus with which to quantify NIK perinulcear staining. Importantly, positive NIK staining was localized predominantly to fibers with reduced CSA (Fig. 3F).

**Figure 4:**
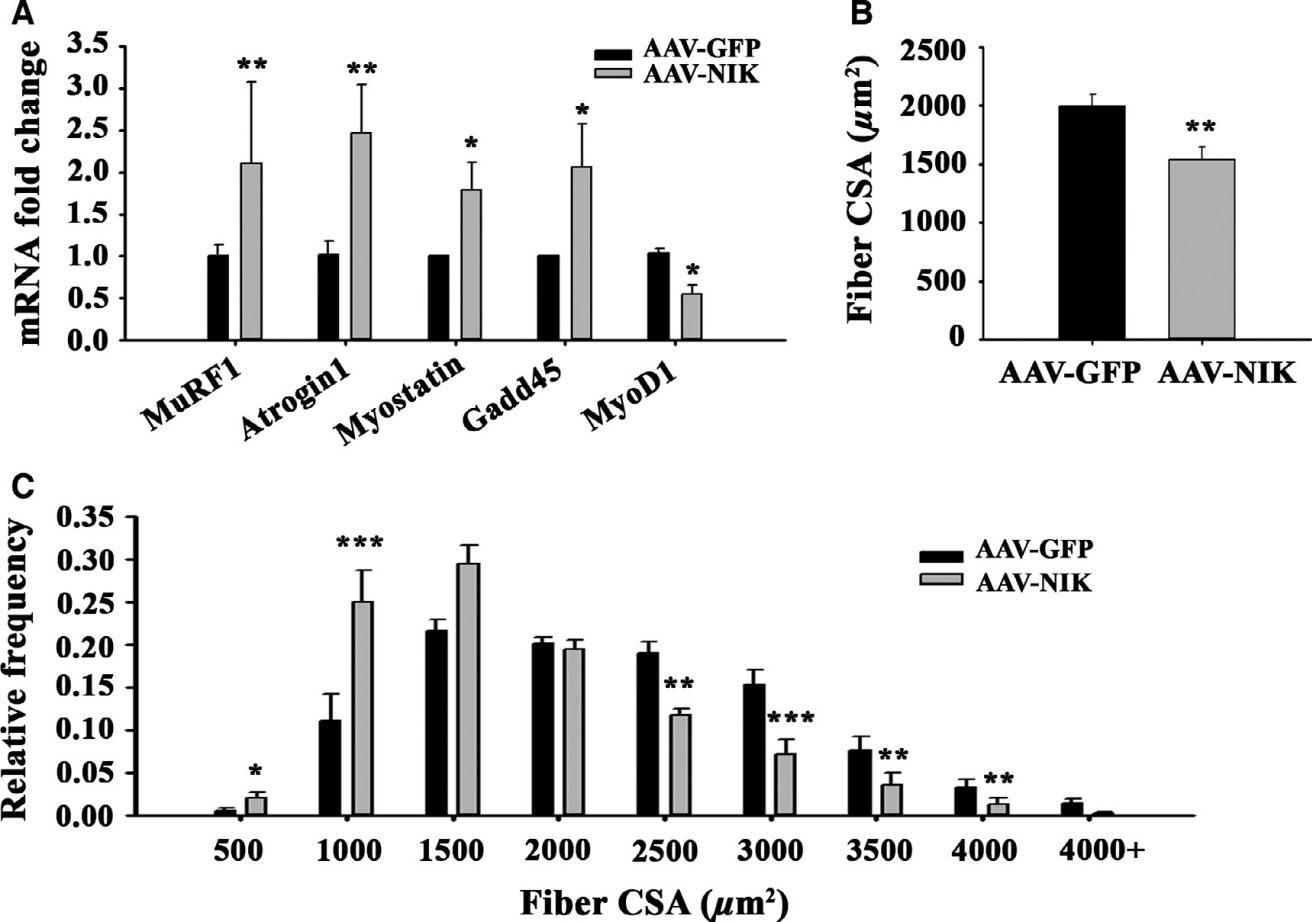
Overexpressing NIK in mouse tibialis anterior induces skeletal muscle fiber atrophy. (A) mRNA expression of various atrogenes and other biomarkers associated with muscle atrophy. (B) Average myofiber cross‐sectional area (CSA) in μm^2^. (C) Histogram distribution of myofiber CSA. Data are presented as mean ± SD. Significantly effect of AAV‐NIK administration: *P < 0.05, **P < 0.01 and ***P < 0.001. *N* = 8 mice/group.

Overexpressing NIK induced significant increases in the mRNA expression of E3 ubiquitin ligases MuRF1 and Atrogin‐1 (Fig. 4A). Myostatin and Gadd45a expression were also increased while mRNA expression of MyoD1 was decreased in AAV‐NIK compared to AAV‐GFP control (Fig. 4A). These changes were associated with a significant 30% decrease in average muscle fiber CSA (Fig. 4B), as well as a significant shift in the relative number of fibers with smaller CSA versus the AAV‐GFP control (Fig. 4C). Taken together, these results suggest that NIK induces skeletal muscle fiber atrophy through up‐regulation of ubiquitin ligases as well as other proteins involved in regulation of skeletal muscle growth.

### Regulation of skeletal muscle protein catabolism by NIK in tissue culture

**Figure 5:**
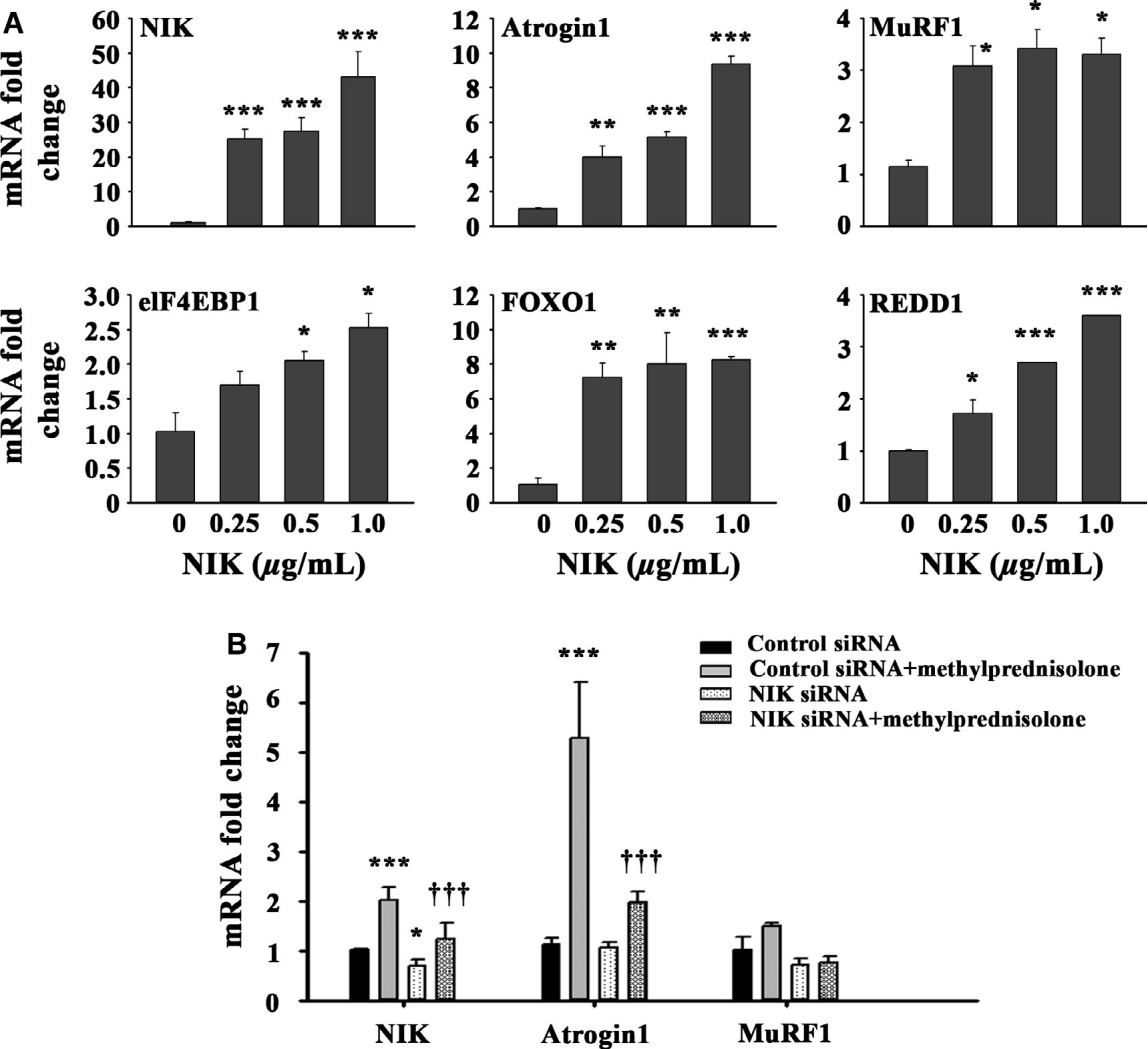
Overexpressing NIK in primary cultures of human myotubes increases transcription of muscle catabolism and atrophy genes. (A) mRNA expression of various atrogenes and other biomarkers associated with muscle atrophy presented as a fold change from control. Significantly effect of NIK overexpression: *P < 0.05, **P < 0.01 and ***P < 0.001. (B) mRNA expression of NIK, Atrogin1 and MuRF1 following administration of methylprednisolone and knockdown of endogenous NIK via siRNA. ***Significant effect of methylprednisolone treatment, *P* < 0.001; *Significant effect of NIK siRNA,* P* < 0.05; ^†††^Significant effect of NIK siRNA,* P* < 0.001. *N* = 4 cell isolates studied in duplicate.

To address further a link between NIK and skeletal muscle catabolism, differentiated primary human myotubes were transiently transfected with increasing concentrations of a NIKwt expression vector (0, 0.25, 0.5 and 1 μg/mL). Dose‐dependent increases in NIK were associated with increased mRNA expression of Atrogin‐1, REDD1 and elF4EBP1 (Fig. 5A), while increases in MuRF1 (3×) and FOXO1 (7–8×) were maximum at the lowest dose of NIKwt vector used. These data demonstrate that increased NIK expression, as observed after GC treatment, is sufficient to dose dependently elevate the expression of various markers of muscle atrophy. In contrast, knocking down NIK with siRNA resulted in a small but statistically significant decrease in basal NIK and significantly blunted the twofold increase in NIK expression following methylprednisolone treatment of primary human myotubes (Fig. 5B). NIK knockdown had no effect on basal Atrogin‐1 mRNA expression but significantly blocked the methylprednisolone‐induced fivefold increase in Atrogin‐1 mRNA expression (Fig. 5B). Under tissue culture conditions used, methylprednisolone induced very little change in MuRF1 gene expression, and MuRF1 expression was unaffected by NIK siRNA knockdown.

## Discussion

The clinical necessity of GC therapy in inflammatory and autoimmune circumstances remains unquestioned even though the adverse effect of glucocorticoids on muscle mass is well characterized. The ability to treat patients with GC and perhaps co‐administer an agent that suppresses or blocks GC‐induced skeletal muscle catabolism has tremendous clinical potential as physicians would not be faced with the challenging side effect of unintended muscle atrophy. Consequently, the mechanism(s) through which glucocorticoids induce skeletal muscle atrophy remains under intense investigation. Here, we have utilized in vivo and in vitro systems, including the direct use of glucocorticoids as well as molecular approaches of overexpression and silencing to provide evidence that increases in NIK, a key upstream kinase that modulates NF‐*κ*B activation (Malinin et al. 1997), are associated with multiple indices of muscle atrophy.

We have reported previously that NIK mRNA levels were significantly elevated following a 6‐day, graded dosing regimen of methylprednisolone in skeletal muscle biopsy specimens collected from human subjects (Urban et al. 2014). Although the increase in NIK mRNA was associated with a corresponding increase in MuRF1 mRNA, this human study could not demonstrate a causal link. We now report that a single, acute exposure to methylprednisolone injected subcutaneously into mice increased NIK mRNA and protein levels in tibialis anterior muscle, consistent with our findings in humans.

NF‐*κ*B‐inducing kinase is a kinase that phosphorylates members of the I*κ*B kinase (IKK) complex, a convergent point for numerous signals that induce NF‐*κ*B activation via both the classical and noncanonical activation pathways (Lin et al. 1998; Xiao et al. 2001; Dejardin et al. 2002; Bonizzi et al. 2004). NIK protein expression is tightly regulated and low basal levels are maintained by regulating turnover by TNF receptor associated factor (TRAF) proteins. In nonstimulated cells, TRAF3 recruits NIK to the complex containing TRAF2 and cellular inhibitors of apoptosis proteins (cIAP1/2). In this complex, NIK undergoes ubiquitination by cIAP1/2 resulting in rapid proteosomal degradation. However, in the presence of NF‐*κ*B noncanonical pathway stimuli, cIAP1/2 ubiquitinates and promotes degradation of TRAF3, thereby releasing NIK from its negative regulation by TRAF3 and leading to its stabilization and accumulation in cells (Vallabhapurapu et al. 2008; Zarnegar et al. 2008). An increased level of NIK induces NIK activation, presumably by autophosphorylation. GC‐mediated muscle wasting also involves activation of the ubiquitin proteasome system and interference of this signaling process protects against GC‐induced muscle atrophy and up‐regulation of the atrogenes Atrogin‐1 and MuRF1 (Bedard et al. 2015; Ochi et al. 2015). Hence, GC‐induced activation of the ubiquitin proteasome may occur through induction of NIK signaling. Although the requirement of NIK is nonredundant in noncanonical pathway activation, NIK activates the canonical pathway by phosphorylating IKK*α*. Thus, constitutive activation of NIK is associated with chronic NF‐*κ*B activation via both canonical and noncanonical pathways. Various genetic or pathological conditions leading to the constitutive activation of NIK have been associated with aging (Kim et al. 2006; Bitar et al. 2010) and diverse chronic inflammatory diseases such as rheumatoid arthritis (Aya et al. 2005), multiple sclerosis, ulcerative sclerosis, chronic hepatitis C and Sjogren's syndrome, hematopoietic tumors (chronic lymphocytic, leukemia, multiple myeloma and cutaneous T‐cell lymphoma) and solid tumors (Rayet and Gelinas 1999; Dhawan et al. 2002; Amiri and Richmond 2005; Aloisi and Pujol‐Borrell 2006; Uno et al. 2014). While we still do not understand the mechanism of action of NIK in skeletal muscle and additional research is necessary to elucidate how NIK integrates into our current understanding of GC‐induced skeletal muscle atrophy, it is noteworthy that these diseases with increased NIK are also associated with severe muscle catabolism.

Overexpression of NIK in the same mouse muscle using a viral vector increased levels of Atrogin‐1 and MuRF1 mRNA, suggesting expression of these atrogenes is linked to NIK. NIK also increased expression of myostatin, which we assessed as a measure of negative control of skeletal muscle protein anabolism (McPherron et al. 1997) and decreased expression of MyoD, which we assayed as a surrogate of satellite cell activity that is tightly correlated with muscle hypertrophy (Ishido et al. 2004). Overexpression of NIK also increased Gadd45a expression, which has been shown to mediate skeletal muscle atrophy in conditions such as fasting, muscle immobilization, and muscle denervation (Caiozzo et al. 2004).

Glucocorticoids therapy for 10 days or overexpressing NIK with adenoviral vectors for 21 days caused a similar decrease in average myofiber CSA and a shift to smaller fiber size (Figs. 2 and 4, respectively) in tibialis anterior muscle. These virtually identical structural changes in the two different animal models occurred despite substantial differences in NIK mRNA induced by glucocorticoid and AAV (2–3 fold induction in Fig. 1A vs. >50‐fold induction in Fig. 3A). Likewise, although nonquantitative, the immunohistochemistry images of NIK staining following glucocorticoid exposure and AAV‐NIK appear comparable in intensity and location of staining (Fig. 1C vs. Fig. 3D) despite the differences in NIK mRNA present based on qRT‐PCR. NIK staining was consistently observed only within the smaller muscle fibers, suggesting that NIK was linked to the observed muscle atrophy. That GFP and NIK staining was observed in 90% and 70% of the muscle fibers, respectively, shows strong infection capacity of the AAV throughout the tibialis anterior muscle.

Overexpressing NIK in relevant primary human myotubes in tissue culture up‐regulated expression of atrogenes associated with muscle catabolism. Additionally, NIK overexpression also induced expression of eIF4EBP1 and REDD1, negative regulators of protein anabolism through the mTORC1 pathway (Hernandez‐Jimenez et al. 2012) In addition to increases in catabolism, increased expression of eIF4EBP1 and REDD1 provides evidence for a suppression of protein synthesis as well, further promoting a net negative protein balance, supportive of the observed muscle atrophy. NIK‐induced expression of FOXO1 may serve as a link to the increased expression of atrogenes MuRF1 and Atrogin‐1, as FOXO1 partly regulates the transcription of the E3 ligases (Lee and Goldberg 2013). Conversely, knocking down endogenous NIK in the same cells with siRNA significantly attenuated GC‐induced NIK and atrogene production. The absence of a robust response of MuRF1 gene expression in response to GC during NIK knockdown differs from the response of Atrogin‐1 in Figure 5B, and makes the response of MuRF1 difficult to interpret. There may be an enhanced sensitivity to elevated NIK by MuRF1 versus Atrogin‐1 as shown in Figure 5A where the lowest dose of NIK expression vector used (0.25 μg/mL) still produced a maximum MuRF1 response. In contrast, a dose‒dependent increase in Atrogin‐1 was evident at the NIK expression vector concentrations used. These results suggest a more complete silencing of endogenous NIK may be required to attenuate or block the GC effect on MuRF1 expression, and indicate potential differences in the sensitivity to NIK by different atrogenes. We did not examine effects of NIK overexpression on myotube size since this measurement to demonstrate induction of atrophy in vitro is relatively uncommon as diameter varies considerably along the length of individual myotubes. While measurement of atrogenes is only correlative of protein degradation, when NIK induction is mitigated by siRNA and the glucocorticoid induction of Atrogin‐1 is severely attenuated, these fingings provide evidence for an integral role of NIK in modulating GC‐induced muscle atrophy through E3 ubiquitin ligases. Taken together, these experimental approaches utilizing NIK overexpression and silencing provide additional support for the suggestion that NIK is causally linked to GC‐induced muscle atrophy.

The immunohistochemical localization of NIK in both the GC and AAV‐NIK experiments to the peri‐nuclear area is consistent with the nuclear function of NIK (Birbach et al. 2004). Likewise, the punctate staining along the inner sarcolemmal membrane of myofibers is consistent with published observations that NIK associates with the outer mitochondrial membrane (Liu et al. 2008). It is noteworthy that interstitial staining of NIK also was observed. While the specific interstitial cell types were not identified and may include satellite cells, resident tissue fibroblasts, or infiltrating immune cells, this finding suggests that glucocorticoids exert their effect on multiple cell types present in skeletal muscle.

A limitation to this study is the difficulty to unambiguously and convincingly demonstrate a causal link between GC, NIK, and skeletal muscle atrophy without the availability of potent and selective pharmacologic inhibitors of NIK or by specifically knocking NIK out in skeletal muscle using recombinant technologies. We have embarked on the latter approach by breeding NIK floxed mice (obtained from Genentech) with a tamoxifen‐inducible ACTA1 cre (Tg(ACTA1‐cre/Esr1*)2Kesr/J; Jackson Labs stock # 025750) so that NIK can be knocked out specifically in limb skeletal muscles. Such an approach has the potential to unambiguously demonstrate a role for NIK in glucocorticoid‐induced skeletal muscle atrophy.

In summary, we provide evidence that induction of NIK is integral in the etiology of GC‐induced muscle atrophy. We are the first to report that upstream regulation of NF‐*κ*B activation occurs in skeletal muscle following glucocorticoid administration. This is highly significant in that it offers novel approaches for exploring glucocorticoid‐induced muscle wasting as well as potentially novel therapeutic approaches that could be used in conjunction with glucocorticoids. In addition, we highlight the immunohistochemical detection of NIK within myofibers following acute glucocorticoid administration. To our knowledge, these are the first data demonstrating the cellular location of NIK within myofibers, and its perinuclear and sarcolemmal location may prove integral to understanding its mechanism of action within muscle. Overexpression of NIK in mouse skeletal muscle and human primary myotubes induced the expression of E3 ubiquitin ligases MuRF1 and Atrogin‐1. Additionally, knockdown of NIK in primary myotubes attenuated glucocorticoid‐mediated increases in Atrogin‐1 expression, providing evidence for a role of NIK in regulating GC‐induced muscle atrophy. Skeletal muscle atrophy is widely appreciated during glucocorticoid administration, and these data highlight the contribution of NIK in its etiology and identify NIK as a potential therapeutic target.

## Conflict of Interest

None declared.
